# Supplementary material for: Rhythmic coordination of hippocampal neurons during associative memory processing
Source: eLife. 2016 Jan 11;5:e09849. doi: 10.7554/eLife.09849 (PMC4718808; doi:10.7554/eLife.09849)
Supplement: Figure 2—source data 1. — The interneurons categorized first by significant spike-phase coherence to a given rhythm and then by coherence during a given performance category (Correct Trials Only, Incorrect Trials Only, All Trials) were further divided by their coherence to each possible combination of the four rhythms examined in this study. For the interneurons that exhibited significant spike-phase coherence to a given rhythm during All Trials, the distribution of their coherence to all possible combinations of rhythms is shown separately for correct and incorrect trials. Interneurons coherent during All Trials often exhibited different profiles of engagement across the four rhythms during correct trials compared to incorrect trials. DOI: http://dx.doi.org/10.7554/eLife.09849.006 [file elife-09849-fig2-data1.docx]

**Figure 2 – Source data 1 | The number of interneurons within each rhythmic category that were coherent to each possible combination of the four rhythms**

|  | **Theta Coherent Category** | | | |
| --- | --- | --- | --- | --- |
| **Rhythmic Combination** | **Correct**  **Only** | **Incorrect**  **Only** | **All Trials**  **Correct** | **All Trials**  **Incorrect** |
| theta | 4 | 2 | 14 | 36 |
| theta, beta | 1 | 0 | 1 | 0 |
| theta, low gamma | 3 | 0 | 2 | 13 |
| theta, high gamma | 10 | 0 | 11 | 10 |
| theta, beta, low gamma | 2 | 0 | 0 | 2 |
| theta, beta, high gamma | 1 | 0 | 0 | 1 |
| theta, low gamma, high gamma | 9 | 0 | 13 | 10 |
| theta, beta, low gamma, high gamma | 10 | 0 | 43 | 12 |

|  | **Beta Coherent Category** | | | |
| --- | --- | --- | --- | --- |
| **Rhythmic Combination** | **Correct**  **Only** | **Incorrect**  **Only** | **All Trials**  **Correct** | **All Trials**  **Incorrect** |
| beta | 2 | 2 | 0 | 0 |
| beta, theta | 2 | 0 | 0 | 0 |
| beta, low gamma | 1 | 0 | 0 | 0 |
| beta, high gamma | 0 | 0 | 0 | 0 |
| beta, theta, low gamma | 2 | 1 | 0 | 1 |
| beta, theta, high gamma | 1 | 0 | 0 | 1 |
| beta, low gamma, high gamma | 1 | 0 | 0 | 0 |
| beta, theta, low gamma, high gamma | 40 | 1 | 13 | 11 |

|  | **Low Gamma Category** | | | |
| --- | --- | --- | --- | --- |
| **Rhythmic Combination** | **Correct**  **Only** | **Incorrect**  **Only** | **All Trials**  **Correct** | **All Trials**  **Incorrect** |
| low gamma | 0 | 1 | 0 | 5 |
| low gamma, theta | 5 | 0 | 0 | 13 |
| low gamma, beta | 1 | 0 | 0 | 0 |
| low gamma, high gamma | 3 | 1 | 1 | 2 |
| low gamma, theta, beta | 2 | 1 | 0 | 1 |
| low gamma, theta, high gamma | 13 | 1 | 9 | 9 |
| low gamma, beta, high gamma | 1 | 0 | 0 | 0 |
| low gamma, theta, beta, high gamma | 21 | 0 | 32 | 12 |

|  | **High Gamma Category** | | | |
| --- | --- | --- | --- | --- |
| **Rhythmic Combination** | **Correct**  **Only** | **Incorrect**  **Only** | **All Trials**  **Correct** | **All Trials**  **Incorrect** |
| high gamma | 2 | 1 | 0 | 3 |
| high gamma, theta | 16 | 1 | 5 | 9 |
| high gamma, beta | 0 | 0 | 0 | 0 |
| high gamma, low gamma | 4 | 0 | 0 | 2 |
| high gamma, theta, beta | 1 | 0 | 0 | 1 |
| high gamma, theta, low gamma | 15 | 1 | 7 | 9 |
| high gamma, beta, low gamma | 1 | 0 | 0 | 0 |
| high gamma, theta, beta, low gamma | 29 | 0 | 24 | 12 |
