# Supplementary material for: Rhythmic coordination of hippocampal neurons during associative memory processing
Source: eLife. 2016 Jan 11;5:e09849. doi: 10.7554/eLife.09849 (PMC4718808; doi:10.7554/eLife.09849)
Supplement: Figure 2—source data 2. — The principal cells categorized first by significant spike-phase coherence to a given rhythm and then by coherence during a given performance category (Correct Trials Only, Incorrect Trials Only, All Trials) were further divided by their coherence to each possible combination of the four rhythms examined in this study. For the principal cells that exhibited significant spike-phase coherence to a given rhythm during All Trials, the distribution of their coherence to all possible combinations of rhythms is shown separately for correct and incorrect trials. DOI: http://dx.doi.org/10.7554/eLife.09849.007 [file elife-09849-fig2-data2.docx]

**Figure 2 – Source data 2 | The number of principal cells within each rhythmic category that were coherent to each possible combination of the four rhythms**

|  | **Theta Coherent Category** | | | |
| --- | --- | --- | --- | --- |
| **Rhythmic Combination** | **Correct**  **Only** | **Incorrect**  **Only** | **All Trials**  **Correct** | **All Trials Incorrect** |
| theta | 178 | 30 | 23 | 41 |
| theta, beta | 13 | 1 | 5 | 3 |
| theta, low gamma | 22 | 3 | 7 | 3 |
| theta, high gamma | 28 | 4 | 10 | 4 |
| theta, beta, low gamma | 4 | 0 | 2 | 0 |
| theta, beta, high gamma | 4 | 0 | 0 | 0 |
| theta, low gamma, high gamma | 10 | 0 | 2 | 0 |
| theta, beta, low gamma, high gamma | 1 | 0 | 2 | 0 |

|  | **Beta Coherent Category** | | | |
| --- | --- | --- | --- | --- |
| **Rhythmic Combination** | **Correct**  **Only** | **Incorrect**  **Only** | **All Trials**  **Correct** | **All Trials Incorrect** |
| beta | 40 | 12 | 0 | 1 |
| beta, theta | 16 | 3 | 2 | 1 |
| beta, low gamma | 2 | 0 | 0 | 1 |
| beta, high gamma | 2 | 0 | 0 | 0 |
| beta, theta, low gamma | 5 | 0 | 1 | 0 |
| beta, theta, high gamma | 4 | 0 | 0 | 0 |
| beta, low gamma, high gamma | 1 | 0 | 0 | 0 |
| beta, theta, low gamma, high gamma | 3 | 0 | 0 | 0 |

|  | **Low Gamma Category** | | | |
| --- | --- | --- | --- | --- |
| **Rhythmic Combination** | **Correct**  **Only** | **Incorrect**  **Only** | **All Trials**  **Correct** | **All Trials Incorrect** |
| low gamma | 41 | 10 | 0 | 2 |
| low gamma, theta | 27 | 5 | 2 | 1 |
| low gamma, beta | 2 | 0 | 0 | 1 |
| low gamma, high gamma | 9 | 2 | 0 | 0 |
| low gamma, theta, beta | 5 | 0 | 1 | 0 |
| low gamma, theta, high gamma | 11 | 0 | 1 | 0 |
| low gamma, beta, high gamma | 1 | 0 | 0 | 0 |
| low gamma, theta, beta, high gamma | 3 | 0 | 0 | 0 |

|  | **High Gamma Category** | | | |
| --- | --- | --- | --- | --- |
| **Rhythmic Combination** | **Correct**  **Only** | **Incorrect**  **Only** | **All Trials**  **Correct** | **All Trials Incorrect** |
| high gamma | 45 | 10 | 2 | 4 |
| high gamma, theta | 35 | 6 | 3 | 2 |
| high gamma, beta | 2 | 0 | 0 | 0 |
| high gamma, low gamma | 9 | 2 | 0 | 0 |
| high gamma, theta, beta | 3 | 0 | 1 | 0 |
| high gamma, theta, low gamma | 12 | 0 | 0 | 0 |
| high gamma, beta, low gamma | 1 | 0 | 0 | 0 |
| high gamma, theta, beta, low gamma | 3 | 0 | 0 | 0 |
